# Sulfotransferase SULT2B1 facilitates colon cancer metastasis by promoting SCD1‐mediated lipid metabolism

**DOI:** 10.1002/ctm2.1587

**Published:** 2024-02-19

**Authors:** Gang Che, Wankun Wang, Jiawei Wang, Cheng He, Jie Yin, Zhendong Chen, Chao He, Xujing Wang, Yan Yang, Jian Liu

**Affiliations:** ^1^ Department of Surgical Oncology The First Affiliated Hospital, School of Medicine Zhejiang University Hangzhou Zhejiang China; ^2^ Center Laboratory, The First Affiliated Hospital, School of Medicine, Zhejiang University Hangzhou Zhejiang China; ^3^ Department of Colorectal Surgery Sir Run Run Shaw Hospital, School of Medicine, Zhejiang University Hangzhou Zhejiang China; ^4^ Department of Thoracic Surgery The First Affiliated Hospital, School of Medicine, Zhejiang University Hangzhou Zhejiang China; ^5^ Department of Colorectal Medicine Zhejiang Cancer Hospital Hangzhou Zhejiang China

**Keywords:** colon cancer, lipid metabolism, metastasis, SULT2B1

## Abstract

Metastasis is responsible for at least 90% of colon cancer (CC)‐related deaths. Lipid metabolism is a critical factor in cancer metastasis, yet the underlying mechanism requires further investigation. Herein, through the utilisation of single‐cell sequencing and proteomics, we identified sulfotransferase SULT2B1 as a novel metastatic tumour marker of CC, which was associated with poor prognosis. CC orthotopic model and in vitro assays showed that SULT2B1 promoted lipid metabolism and metastasis. Moreover, SULT2B1 directly interacted with SCD1 to facilitate lipid metabolism and promoted metastasis of CC cells. And the combined application of SCD1 inhibitor CAY with SULT2B1‐ konockout (KO) demonstrated a more robust inhibitory effect on lipid metabolism and metastasis of CC cells in comparison to sole application of SULT2B1‐KO. Notably, we revealed that lovastatin can block the SULT2B1‐induced promotion of lipid metabolism and distant metastasis in vivo. Further evidence showed that SMC1A transcriptionally upregulated the expression of SULT2B1. Our findings unveiled the critical role of SULT2B1 in CC metastasis and provided a new perspective for the treatment of CC patients with distant metastasis.

## Graphical abstract

1. SULT2B1 promoted lipid metabolism and metastasis of colon cancer.

2. SULT2B1 interacted with SCD1 to facilitate lipid metabolism and metastasis.

3. SMC1A transcriptionally upregulated the expression of SULT2B1 in colon cancer.

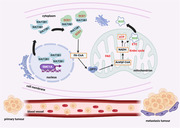

## 1 INTRODUCTION

Colon cancer (CC) is a prevalent and deadly malignancy worldwide,^1^ with an increasing incidence observed among patients under 50 years old.^2^ Epidemiological data reveal that 20% of colon cancer patients are diagnosed with distant metastasis, and over 50% of CC patients develop distant metastasis within 5 years of primary tumour diagnosis,^3^ which has been considered to be responsible for at least 90% of CC‐associated mortality. However, the molecular mechanisms driving CC metastasis have not been fully elucidated. Thus, it is critical to comprehend the underlying regulatory programmes that fuel CC metastasis to improve patients’ outcomes. To achieve this goal, further research is required.

Lipid metabolism plays a significant role in the proliferation,^4^, ^5^, ^6^, ^7^ metastasis^8^, ^9^, ^10^ and drug therapy^11^ of tumour cells, making it an attractive target for inhibiting these activities in tumours. Inhibition of key enzymes of lipid metabolism is considered a prospective method to reduce tumour metastasis.^12^, ^13^, ^14^, ^15^, ^16^, ^17^, ^18^, ^19^ While several clinical trials have explored the inhibition of key enzymes of lipid metabolism in cancers, there are currently no positive clinical trials on CC in this regard. Therefore, there is a pressing need to identify potential targets in lipid metabolism that could effectively inhibit distant metastasis of CC.

In the process of sulfate conjugation, sulfotransferase enzymes are crucial participants, facilitating the conjugation of hormones, neurotransmitters, drugs and xenobiotic compounds.^20^ SULT2B1, a member of the sulfotransferase family, also referred to as sulfotransferase family 2B member 1, has been associated with the progression of multiple cancer types, including prostate cancer,^21^ gastric cancer^22^ and oesophagus cancer.^23^ Recent research has suggested that high expression of SULT2B1 could potentially accelerate the progression of liver cancer by impacting the tumour microenvironment.^24^ Nevertheless, the effect of SULT2B1‐mediated lipid metabolism on the progression of CC remains unclear.

Herein, we identified SULT2B1 as a novel metastasizing tumour marker for CC, which promoted lipid metabolism and metastasis capacity of CC cells by directly interacting with stearoyl‐CoA desaturase (SCD1). And lovastatin can block SULT2B1‐induced promotion effect of lipid metabolism and distant metastasis. In addition, we demonstrated that structural maintenance of chromosomes 1A (SMC1A) transcriptionally upregulated SULT2B1 expression. Our findings unveiled the critical role of SULT2B1 in CC metastasis and provided a new perspective for the treatment of CC with distant metastasis.

## 2 RESULTS

### 2.1 SULT2B1 was associated with poor prognosis and metastasis of CC

**FIGURE 1:**
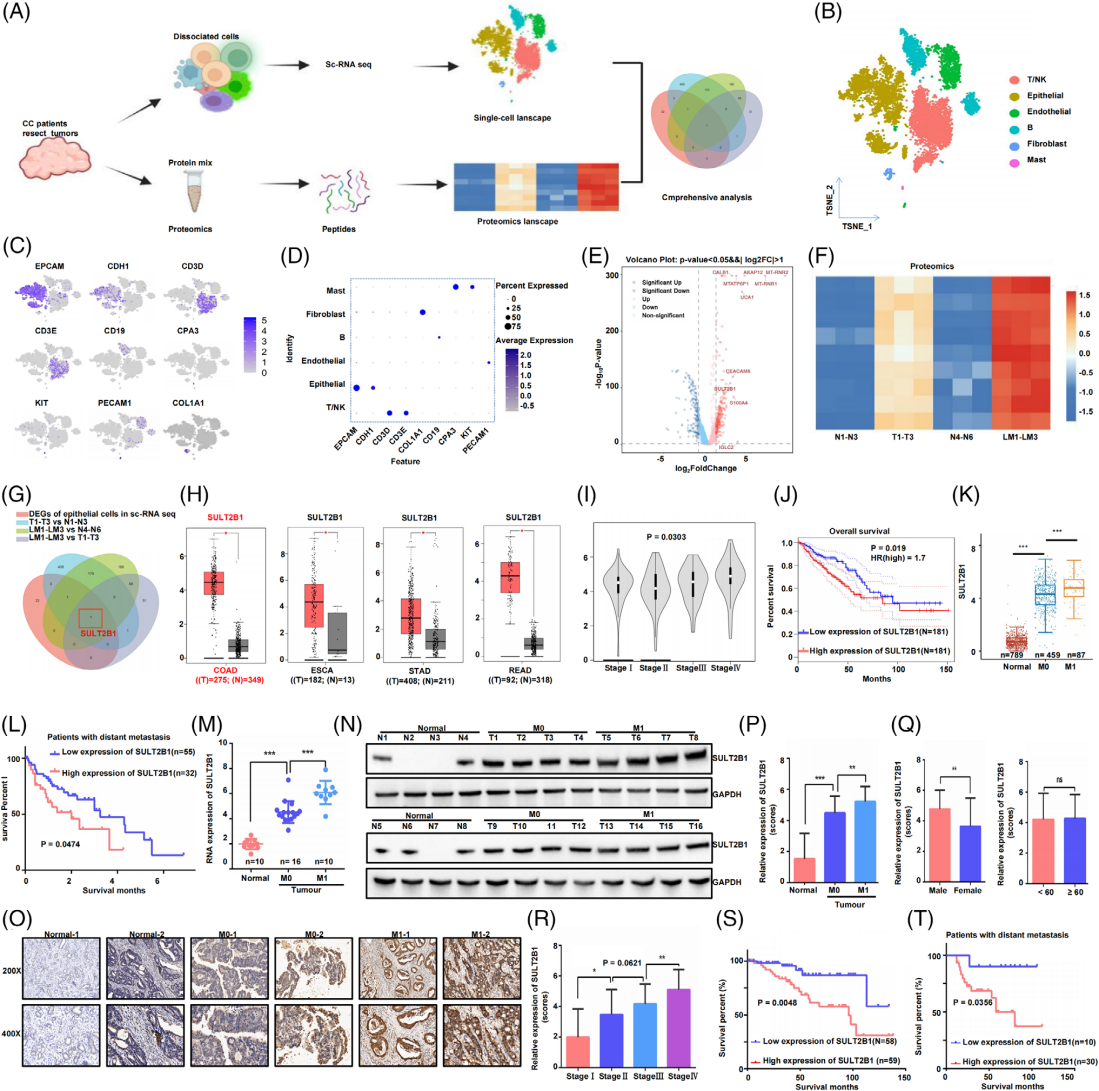
High expression of SULT2B1 was associated with poor prognosis of CC patients and metastasis. (A) Schematic illustration of identifying metastatic marker. (B) TSNE plot of complete cell clusters from 12 samples. (C) Feature plot and (D) dot plot of marker genes sorted by cell type. (E) Volcano plot of DEGs of epithelial cells in the single‐cell sequencing. (F) Heatmap of DEPs of proteomics. (G) Venn plot of comprehensive analysis of sc‐seq and proteomics. (H) SULT2B1 expression level in ESCA, STAD, COAD, READ. (I) SULT2B1 expression in CC by cancer stage. (J) The overall survival of CC patients by SULT2B1 levels. (K) SULT2B1 expression level in patients by metastatic state. (L) The overall survival of metastatic CC patients by SULT2B1 levels. Results of Figure 1H–L are based on data in TCGA. (M–P) SULT2B1 expression in patients by (m) RT‐QPCR, (n)WB and (O and P) IHC. (Q and R) SULT2B1 expression level by sex, age and stage of patients via IHC. (S) The overall survival of 117 CC patients by SULT2B1 levels. (T) The overall survival of 40 metastatic CC patients by SULT2B1 levels.

To analyse metastatic markers of CC, scRNA‐seq data and proteomics were utilised (Figure 1A). Here, scRNA‐seq data of four normal, five primary tumour and three liver metastasis tissues were obtained from GEO database, which were successfully identified to six major cell clusters (Figure 1B), and the expression pattern of classic markers associated with these cells remained in accordance with the annotation (Figure 1C,D).

Next, we analysed the differential expression genes (DEGs) of epithelial cells in scRNA‐seq, and identified 27 upregulated DEGs (log2[fold change] as >2 or <−2 and *p*‐value <0.05 were set as the thresholds) in CC patients with liver metastasis compared to normal or primary tumour, which were visualised via a Volcano plot (Figure 1E). Meanwhile, we collected 12 fresh postoperative specimens at the First Affiliated Hospital of Zhejiang University for proteomics, identifying nine upregulated differential expression proteins (DEPs, the thresholds of fold change was set as >1.2 or <0.83) in CC patients with liver metastasis compared to normal or primary tumour.(Figure 1F). By comprehensive analysis of DEGs of epithelial cells in scRNA‐seq and DEPs of proteomics, we notably found SULT2B1 was the only one metastatic marker (Figure 1G). And we also found that SULT2B1 expression was significantly higher in tumour tissues of digestive tract cancers in the TCGA database (Figure 1H). The expression of SULT2B1 exhibited a significant correlation with tumour stage (Figure 1I) and prognostic in CC (Figure 1J). Furthermore, in patients with distant metastasis, SULT2B1 expression was much higher (Figure 1K), and high expression of SULT2B1 was associated with worse prognosis (Figure 1L). Hence, these findings suggested that SULT2B1 could function as a potent oncogene, enhancing metastatic potential.

To confirm the results obtained from the database analysis, patients' tissues, pathological sections and clinicopathological data were collected. Our results showed that SULT2B1 expression in tumour tissues was higher than that in patients without distant metastasis or normal tissues (Figure 1M–P). Moreover, high expression of SULT2B1 was significantly associated with sex, stage and poor prognosis, but not with age (Figure 1Q–S). Importantly, in patients with distant metastases, elevated levels of SULT2B1 meant worse survival rates (Figure 1T). The collective findings suggested a correlation between elevated SULT2B1 expression and unfavourable prognosis among patients diagnosed with CC, particularly in cases involving distant metastasis.

### 2.2 SULT2B1 promoted migration and metastasis of CC

**FIGURE 2:**
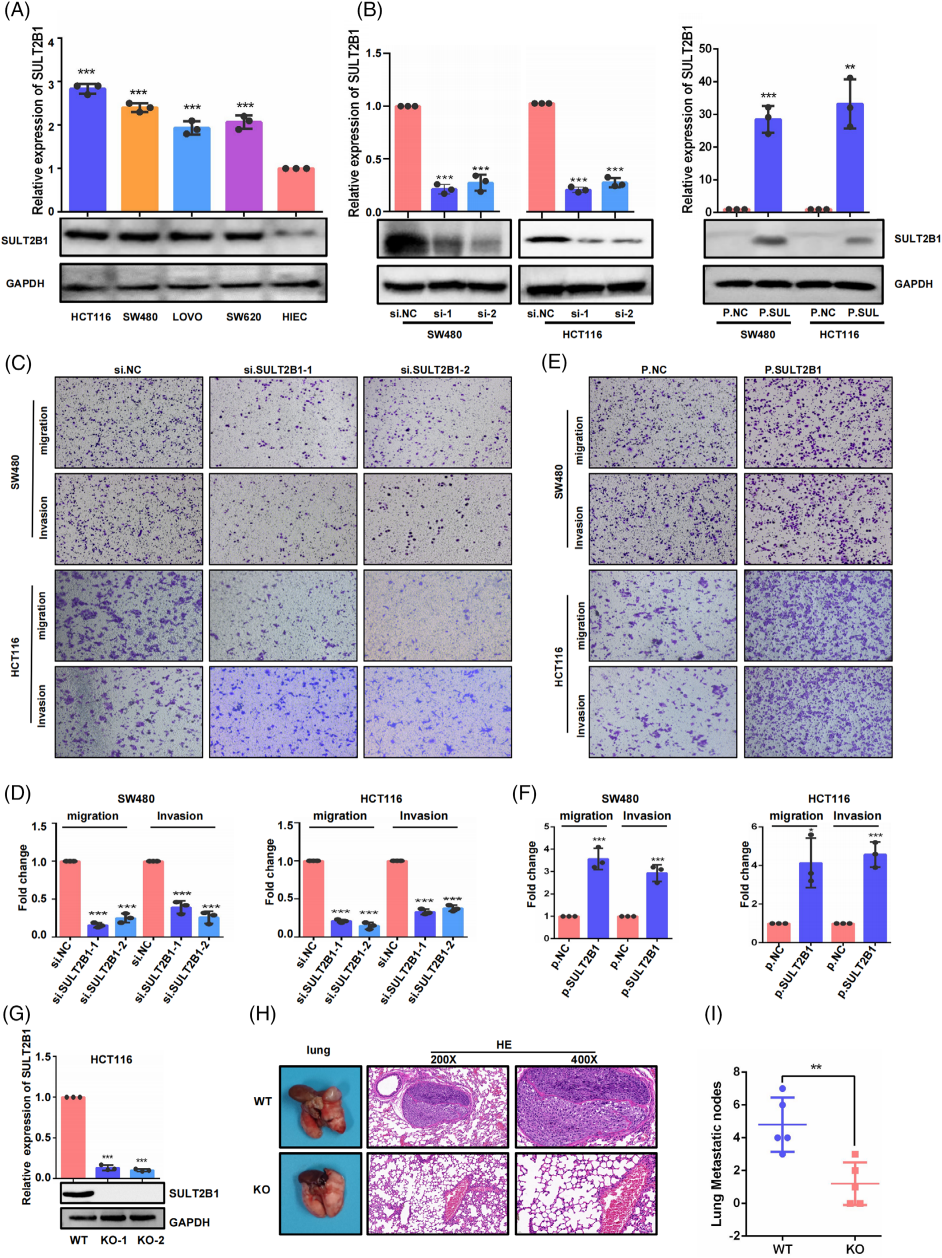
SULT2B1 promoted metastatic capacity in CC. (A) SULT2B1 expression in CC cell lines and normal intestinal epithelial cells (HIEC). (B) The intervention effects of SULT2B1 knockdown and overexpression were evaluated using RT‐QPCR and WB. (C–F) The capacity of migration and invasion in HCT116 and SW480 cells were tested after SULT2B1 knockdown or SULT2B1 overexpression. (G) SULT2B1‐KO effects were evaluated by RT‐QPCR and WB. (H) Representative image of lung in mice injected with indicated cancer cells via tail vein, and HE staining of lung tissues. (I) The number of lung metastatic nodules. KO, knockout; WT, wild type.

In addition, we found that SULT2B1 expression in CC cell lines was generally higher than that in normal intestinal epithelial cells (HIEC cells), particularly in SW480 and HCT116 cell lines (Figure 2A). siRNA and overexpression plasmid were used to knockdown or overexpress SULT2B1 (Figure 2B). And it was found that SULT2B1 knockdown significantly inhibited migration and invasion of SW480 and HCT116 cells, while overexpression of SULT2B1 can obviously promote these effects (Figure 2C–F).

To elucidate the in vivo role of SULT2B1, SULT2B1‐KO cells were established. Subsequently, these cells were intravenously inoculated into mice to investigate their propensity for lung metastasis. The finding revealed a significant reduction in lung metastases of CC cells following SULT2B1‐KO induction (Figure 2G–I). Collectively, these findings suggested that SULT2B1 played a significant role in inducing the metastatic capacity of CC.

### 2.3 SULT2B1 induced lipid metabolism of CC

**FIGURE 3:**
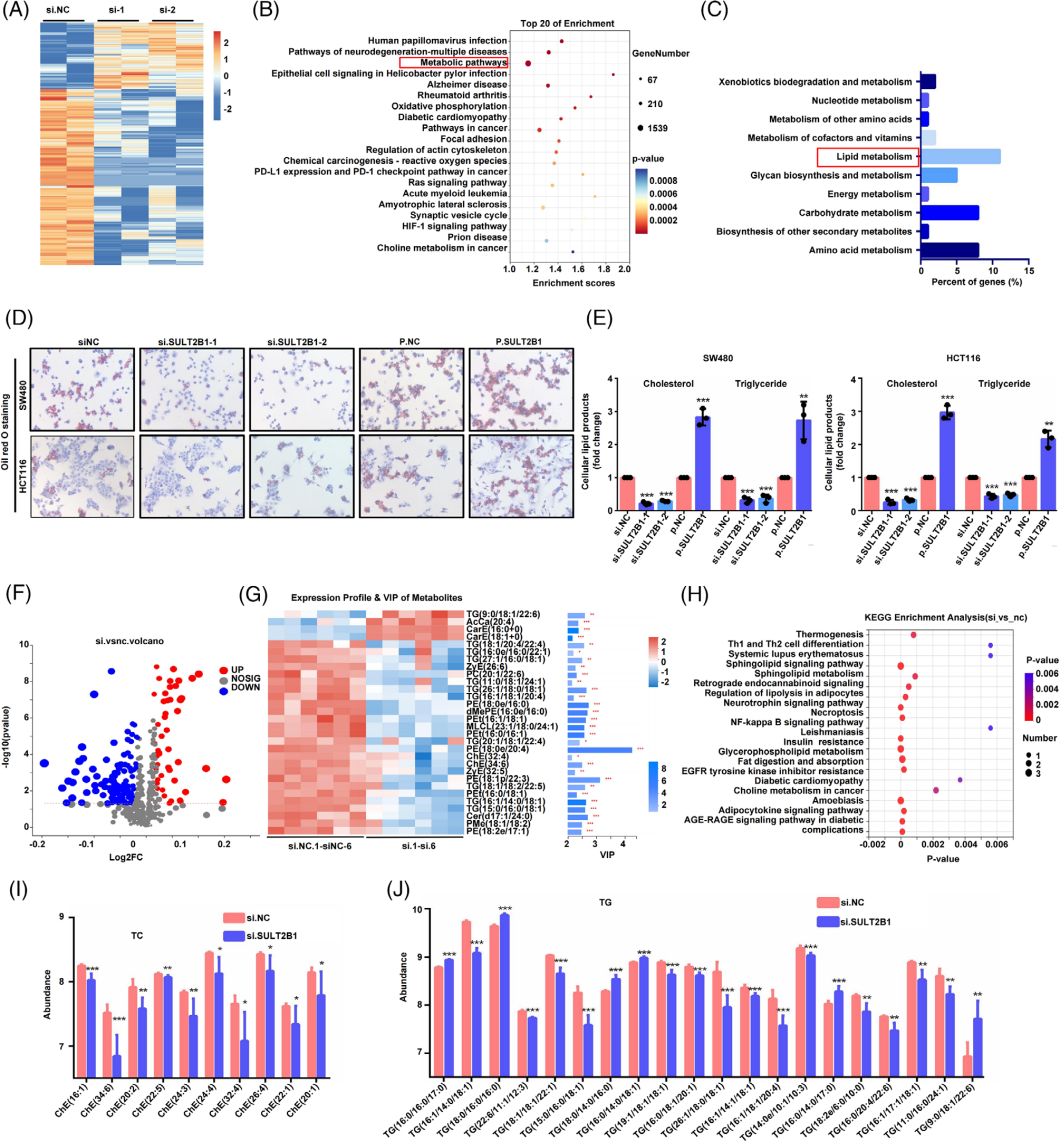
SULT2B1 facilitated lipid metabolism in CC. (A) Heatmap plot of DEGs in RNA‐seq. (B) Bubble plot of KEGG enrichment results. (C) Bar plot presents the metabolic module of KEGG classification results. (D and E) Lipid metabolism activities of CC cells were evaluated by Oil red O staining, TG and TC assays after SULT2B1 knockdown. (F) Volcano plot visualised differential metabolites of non‐target lipomics. (G) Heatmap presents top 30 metabolites. (H) Bar plot displays pathway enrichment of differential metabolites. (I and J) TC and TG accumulation (top 20 TG are shown) are displayed after SULT2B1 knockdown.

To investigate the mechanism by which SULT2B1 affected the metastatic capacity of CC, we performed RNA‐seq analysis on HCT116 cells (Figure 3A). Interestingly, metabolic pathway was found to be the top one KEGG enrichment pathway of DEGs in RNA‐seq (Figure 3B). Further, lipid metabolism was found to be the most significant metabolic pathway, which was also the only pathway SULT2B1 implicated (Figure 3C). Moreover, previous studies have reported that SULT2B1 plays a key role in producing cholesterol sulfotransferase,^25^ and its mutation can lead to altered epidermal cholesterol metabolism.^26^ Therefore, we inferred that SULT2B1 might facilitate the metastasis capacity of CC through lipid metabolism. Our results showed that SULT2B1 can significantly regulate lipid accumulation in CC cells (Figure 3D,E). And on‐target lipidomics further confirmed that SULT2B1 knockdown can reduce lipid accumulation of HCT116 cells (Figure 3F–J and Figure S1). Ultimately, we demonstrated that SULT2B1 significantly induced lipid metabolism in CC cells.

### 2.4 SULT2B1 co‐interacted with SCD1 to activate lipid metabolism promoting metastasis of CC

**FIGURE 4:**
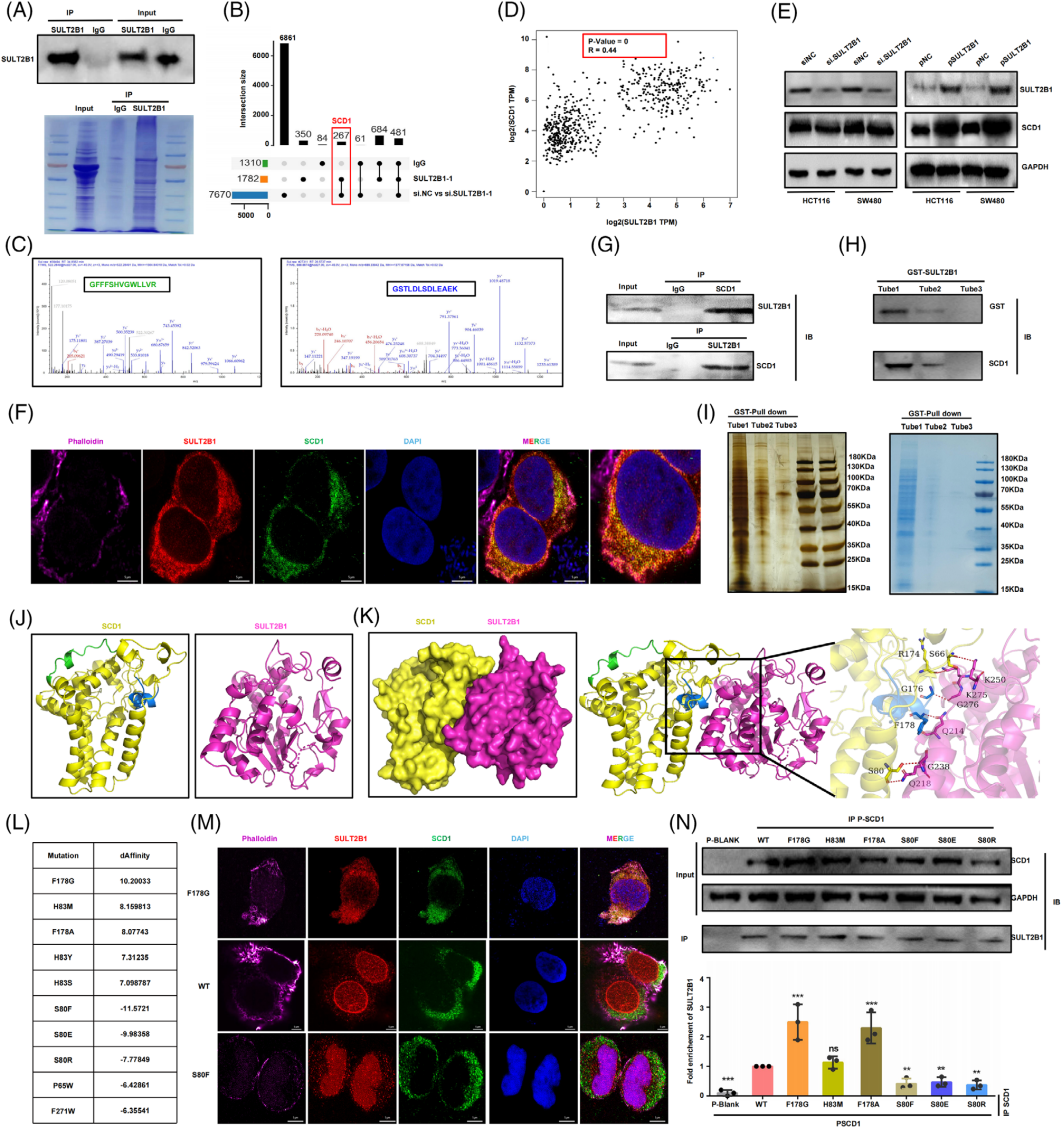
SULT2B1 co‐interacted with SCD1 in HCT116 cells. (A) IP‐MS was performed to identify SULT2B1 co‐interaction protein. (B) Upset plot visualised intersections of IP‐MS and DEGs of RNA‐seq. (C) SCD1 peptides were immunoprecipitated by SULT2B1. (D) Correlation plot between SULT2B1 and SCD1 in TCGA data. (E) SULT2B1 expression can significantly affect the expression level of SCD1. (F–I) The interaction of SULT2B1 and SCD1 in HCT116 cells were detected using IF, Co‐IP and pull‐down assays (Tube 1−3: protein eluted from GST protein purification column). (J) 3D structure of SULT2B1 and SCD1. (k) Interaction model of SULT2B1 and SCD1. (I) The amino acid mutations in SCD1 that would affect binding affinity between SCD1 and SULT2B1 by virtual residue scanning calculation. (m and n) The interaction of SULT2B1 and SCD1 in HCT116 cells were detected using IF (m) and Co‐IP (n) after amino acid mutations in SCD1.

To elucidate the molecular mechanism underlying the effect of SULT2B1 on lipid metabolism and metastasis of CC cells, immunoprecipitations mass spectrometry (IP‐MS) was performed to identify SULT2B1‐interacting proteins in HCT116 cells (Figure 4A). We found that SCD1 specifically co‐precipitated with SULT2B1 (Figure 4B‐C), and there existed a notably strong positive correlation between the expression of SCD1 and SULT2B1 in CC patients (Figure 4D). Meanwhile, the expression of SCD1 was also positively affected by SULT2B1 in CC cells (Figure 4E). To further confirm this interaction, we used immunofluorescence (IF), co‐IP and pull‐down assays, which clearly exhibited a favourable indication of the interaction between SULT2B1 and SCD1 within HCT116 cells (Figure 4F–I). 3D structures of SULT2B1^27^ and SCD1^28^ were downloaded from the PDB protein database^29^ (Figure 4J), and their interaction model was predicted by HDOCK,^30^ which was visualised via PYMOL software^31^ (Figure 4K). To identify mutations in SCD1 that affect the binding affinity between SCD1 and SULT2B1, we conducted virtual residue scanning calculations and found that Phenylalanine 178th, Serine 80th and Histidine 83rd played an essential role in this interaction, which was further confirmed via IF and co‐IP (Figure 4L–N and Figure S2). In addition, it was found that site mutation can obviously regulate lipid metabolism and metastasis in CC cells (Figure S3).

**FIGURE 5:**
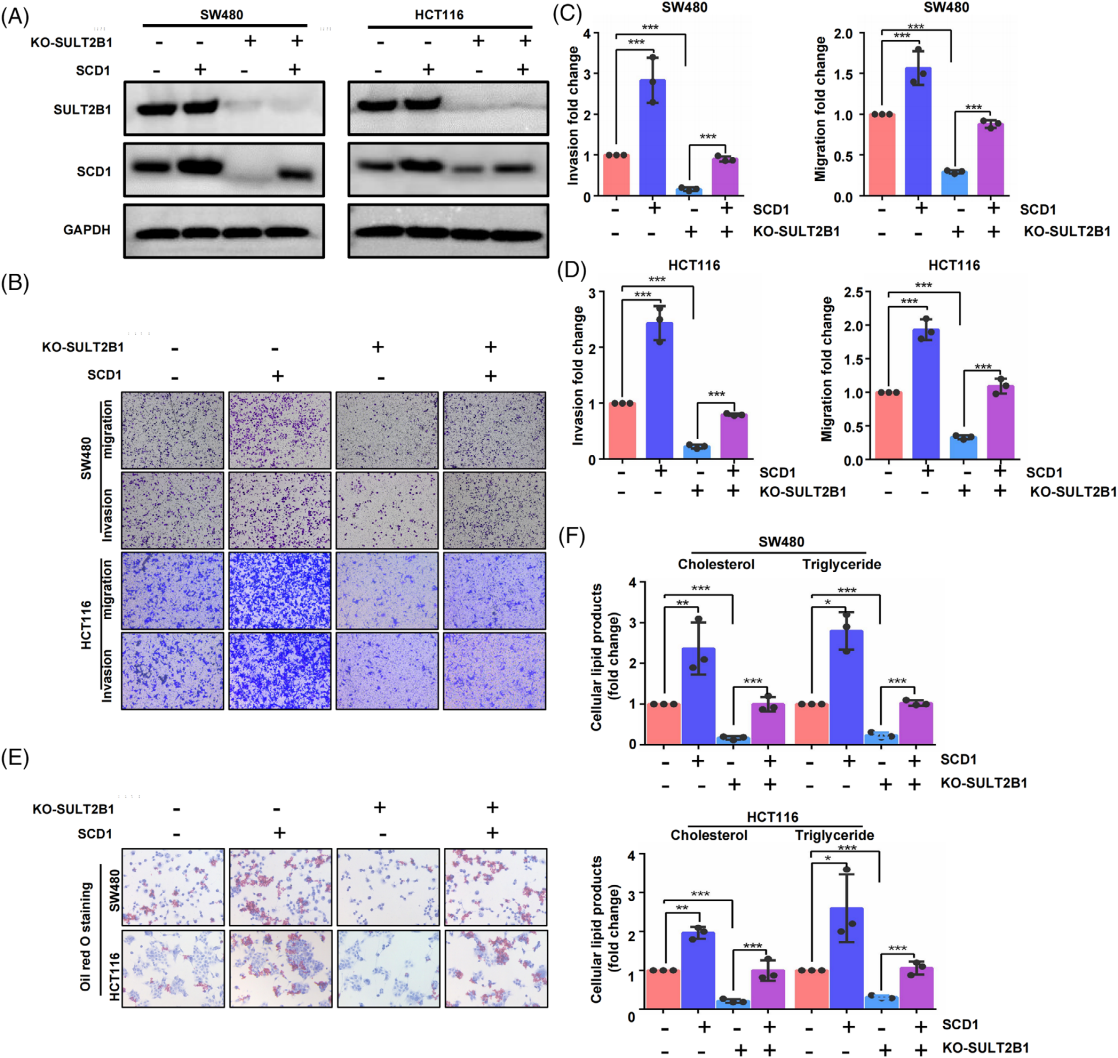
SULT2B1 induced lipid metabolism promoting metastasis in CC via directly upregulating SCD1. (A) WB visualised the intervention effects of SULT2B1 knockout and overexpression of SCD1. (B–D) SULT2B1‐KO can significantly inhibit the capacity of migration and invasion in HCT116 and SW480 cells, whereas SCD1 overexpression can block these events. (E and F) SULT2B1‐KO can significantly reduce the lipid metabolism in HCT116 and SW480 cells, while SCD1 overexpression can interdict them.

Then, gene recovery experiments were performed to verify that SULT2B1 induced lipid metabolism, promoting the metastasis of CC cells through SCD1. The results showed that SULT2B1‐KO could inhibit the metastasis capacity in CC cells, whereas SCD1 overexpression could block these events (Figure 5A–D). Concurrently, SCD1 overexpression could block the effects of lipid metabolism caused by SULT2B1‐KO (Figure 5E,F). Overall, our findings confirmed the significant role of SULT2B1 in inducing the metabolic activity of CC and promoting their metastasis by directly co‐interacting with SCD1.

### 2.5 SMC1A upregulated SULT2B1 expression in CC

**FIGURE 6:**
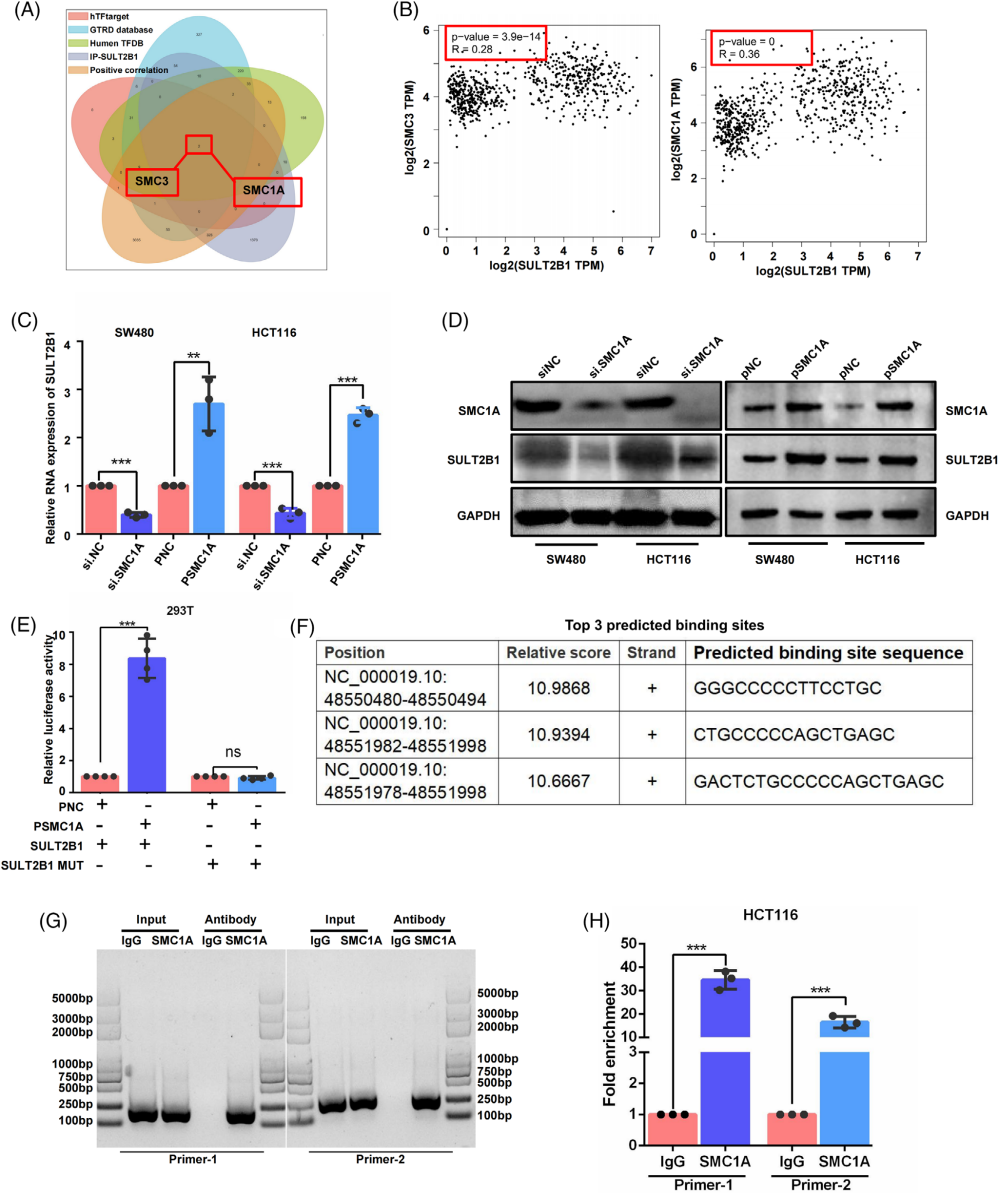
SMC1A upregulated SULT2B1 expression in CC. (A) Venn plot of the transcription factors that may regulate SULT2B1, the molecules that were significantly positively related to SULT2B1, and the molecules that interacted with SULT2B1. (B) Correlation plot between SULT2B1 and SMC3, SMC1A. (C and D) SULT2B1 expression was measured after knockdown or overexpression of SMC1A. (E) Promoter activity of SULT2B1 was evaluated by double luciferase reporter gene experiment after SMC1A overexpression; SULT2B1 MUT: mutation of SULT2B1 promoter, (F) Top three predicted binding site sequences predicted by human TFDB. (G and H) Chip‐PCR confirmed that SMC1A can directly bind DNA sequence of the SULT2B1 gene. (G) DNA gel electrophoresis of chip‐PCR. (H) RT‐QPCR of chip‐PCR.

The results above demonstrated that SULT2B1 activated lipo‐metabolism properties and metastasis of CC by directly co‐interacting with SCD1. To identify the mechanism of SULT2B1 overexpression in CC, we analysed the transcription factors regulated SULT2B1 through hTFtarget,^32^ human TFDB^33^ and GTRD database,^34^ the molecules positively related to SULT2B1 via Assistant for Clinical Bioinformatics,^35^ and the molecules interacted with SULT2B1 by IP‐MS. This led us to identify two transcription factors (SMC3 (structural maintenance of chromosomes 3) and SMC1A) that might upregulate the expression of SULT2B1 (Figure 6A,B). Subsequently, we discovered that knockdown or overexpression of SMC1A affected SULT2B1 expression while SMC3 did not (Figure 6C,D and Figure S4). Meanwhile, it was demonstrated that SMC1A overexpression can significantly activate SULT2B1 promoter activity (Figure 6E). Additionally, CHIP‐PCR investigation indicated that SMC1A could directly bind DNA sequence of the SULT2B1 gene (Figure 6F–H). In conclusion, SMC1A transcriptionally upregulated SULT2B1 expression in CC.

### 2.6 SULT2B1 induced lipid metabolism to promote metastasis through SCD1 in vivo

**FIGURE 7:**
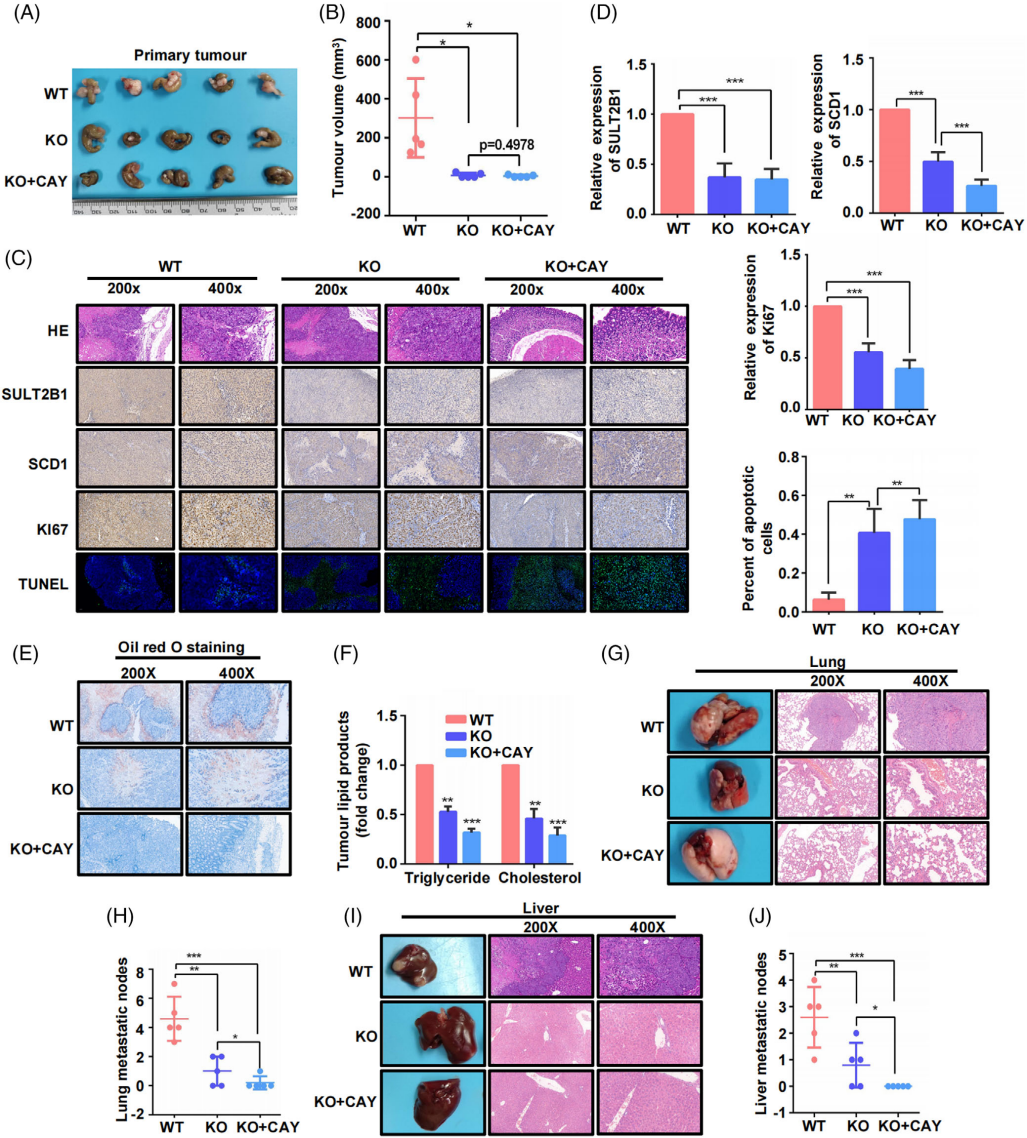
SULT2B1 knockout decreased metastasis capacity of CC cells in vivo. (A and B) Primary tumour and tumour volume in mice among the three groups. (C and D) HE staining, IHC of SULT2B1, SCD1and Ki67, and TUNEL assay of primary tumour in the three groups. (E and F) Lipid accumulation of primary tumour in three groups. (G–J) Lung and liver metastasis of the three groups.

The CC orthotopic model was established to further investigate the findings above. Results showed that SULT2B1‐KO significantly inhibited tumour proliferation, induced apoptosis of tumour cells and repressed lipid metabolism activities of tumours in situ, along with decreased SCD1 expression (Figure 7A–F). Importantly, we demonstrated that SULT2B1‐KO can obviously decrease lung and liver metastasis of CC cells (Figure 7G–J). Moreover, the combined application of SCD1 inhibitor CAY with SULT2B1‐KO demonstrated a more robust inhibitory effect on both the metastasis and lipid metabolism of CC cells in comparison to the sole application of SULT2B1‐KO (Figure 7).

**FIGURE 8:**
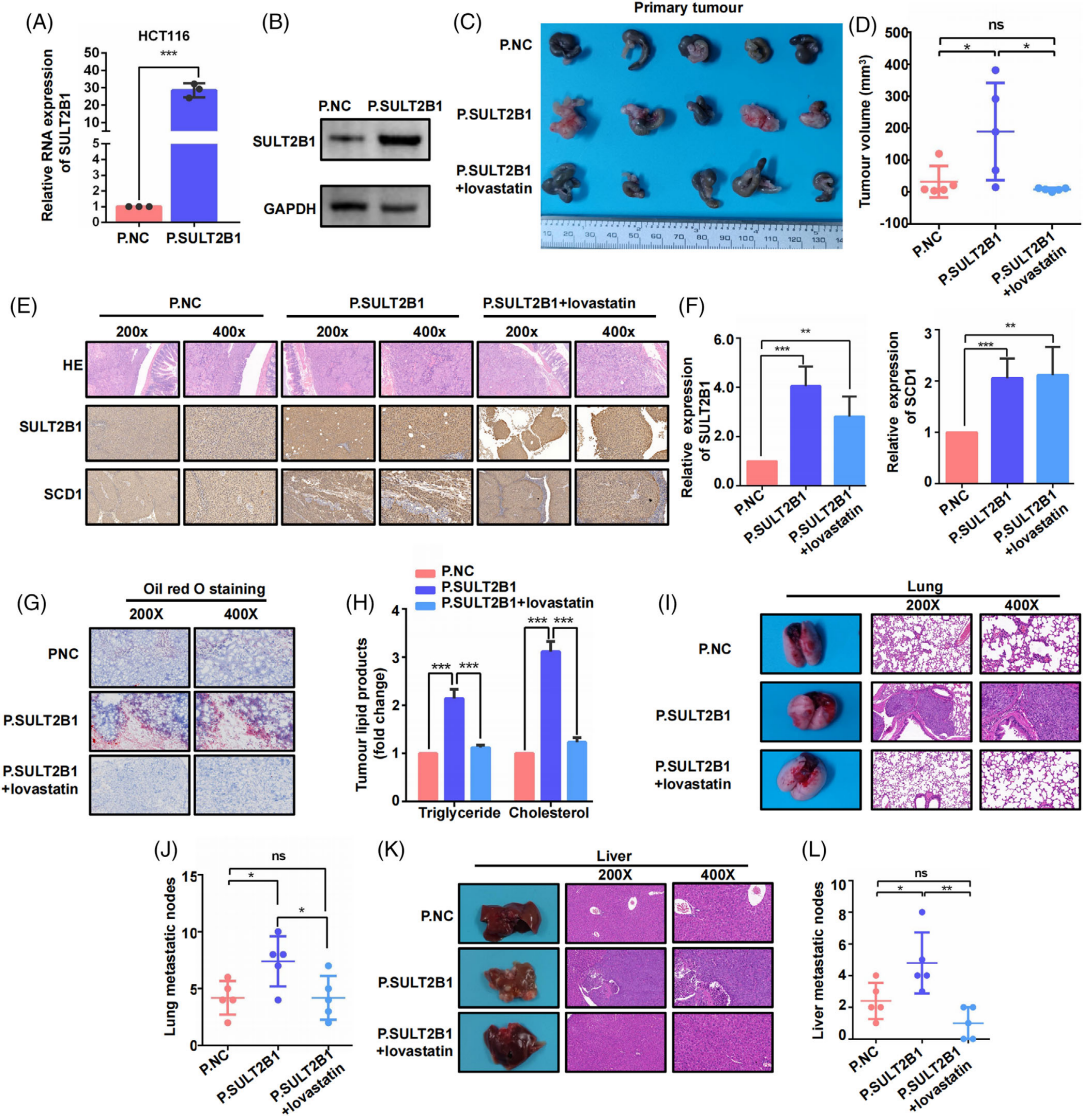
Lovastatin‐blocked SULT2B1 induced lipid metabolism and metastasis in CC cells. (A and B) RT‐QPCR and WB evaluation of SULT2B1‐overexpression stable cell of HCT116. (C and D) Primary tumour and tumour volume in mice among the three group. (E and F) HE staining, and IHC of SULT2B1, SCD1 of primary tumour in the three groups. (G and H) Lipid accumulation of primary tumour in three groups. (I–L) Lung and liver metastasis of the three groups.

Additionally, a stable HCT116 SULT2B1‐overexpression cell was established (Figure 8A,B). In vivo experiments demonstrated that the overexpression of SULT2B1 facilitated tumour proliferation, increased SCD1 expression, facilitated lipid metabolism activities and promoted distant metastasis (Figure 8). Meanwhile, by Transwell assays, it was found that lovastatin can obviously suppress SULT2B1‐induced metastatic activity (Figure S5). In vivo, it was discovered that lovastatin can block the promotion effect of distant metastasis as well as lipid metabolism activities by SULT2B1 overexpression (Figure 8). In conclusion, the results confirmed that SULT2B1 regulated SCD1 in a positive feedback manner to induce lipid metabolism, promoting metastasis of CC.

**FIGURE 9:**
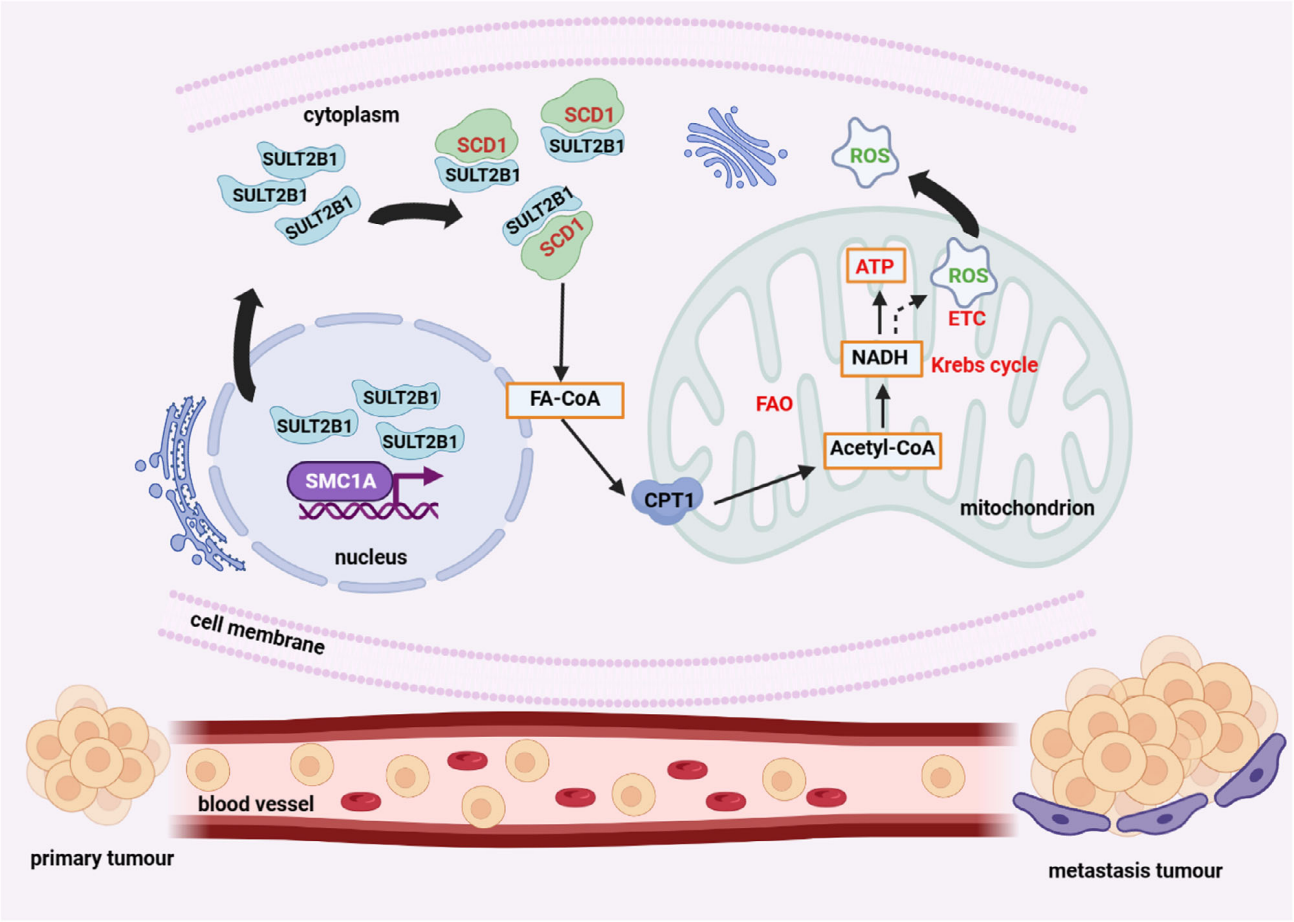
The abstract diagram interpreting the mechanisms that SULT2B1 facilitated in colon cancer metastasis by promoting SCD1‐mediated lipid metabolism.

## 3 DISCUSSION

The clinical outcomes for CC patients continue to be unsatisfactory as effective molecularly targeted medications are still unavailable. Lipid metabolism significantly influences the occurrence and progression of CC,^36^, ^37^, ^38^ but the underlying mechanisms are not fully understood.

In this study, sc‐RNA seq and proteomics were used to identify the metastatic maker of CC. Despite the sc‐RNA sequencing data retrieved from the GEO database lacking corresponding information on adjacent cancerous tissues, our analysis revealed a notably elevated expression of SULT2B1 within metastatic CC tumour tissues. Additionally, its expression levels were positively related with the stages of CC. High expression of SULT2B1 was found to be related to poor prognosis in CC patients with distant metastasis. Interestingly, SULT2B1 is highly expressed in prostate cancer^39^, ^40^ and gastric cancer.^22^ Despite this, the reason for the high expression of SULT2B1 in tumours has not been established. In this study, we demonstrated that SMC1A transcriptionally upregulated SULT2B1 expression in CC cells. In vivo and in vitro experiments additionally demonstrated the depletion of SULT2B1 inhibiting the metastatic phenotypes of CC. These data indicated that SULT2B1 might act as a potential adjuvant marker for treatment of CC, especially for those with distant metastasis.

KEGG pathway enrichment of RNA‐seq indicated that SULT2B1 predominantly participated in the lipid metabolism pathway. Given the critical role of abnormal metabolism in cancer progression,^41^, ^42^, ^43^ and the involvement of SULT2B1 in lipid metabolism,^25^, ^26^ we hypothesised that SULT2B1 may promote metastasis in CC through its participation in lipid metabolism. Our results demonstrated that SULT2B1 in CC cells regulated the intracellular lipid product levels, suggesting that SULT2B1 promoted lipid metabolism in CC. It was well known that lipo‐metabolism in cancer cells is closely associated with critical lipogenic enzymes, including fatty acid synthase (FASN),^7^ acetyl‐CoA carboxylase alpha (ACC1),^44^, ^45^ SCD1,^46^, ^47^ SERPINE1 MRNA binding protein 1 (SERBP1)^48^ and ATP citrate lyase (ACLY).^49^ Therefore, we speculated that SULT2B1 might promote tumour cell lipid metabolism and metastasis by regulating a key enzyme in lipo‐metabolism pathway. To test this hypothesis, we performed co‐IP and protein LC‐MS analysis, which revealed that SULT2B1 directly interacted with SCD1 in HCT116 cells.

To investigate whether amino acids affect the regulation between SULT2B1 and SCD1, SCD1 amino acid mutation scanning was performed. The results revealed that Phenylalanine 178th, Serine 80th and Histidine 83th had a vital effect on affinity. Meanwhile, it was demonstrated that site mutation of SCD1 can obviously regulate lipid metabolism and metastasis in CC cells in the in vitro experiments. Hence, it was suggested that the interaction between SULT2B1 and SCD1 predominantly regulated lipid metabolism and metastasis in CC cells, with SCD1 also playing a participatory role in this process. Regrettably, due to time and budgetary constraints, we were unable to conduct experiments in animal models to fully elucidate the role of the mutation at the interaction site between SULT2B1 and SCD1 in lipid metabolism and metastasis. In addition, our finding validated that SCD1 could block the inhibition of metastasis capacity and lipid metabolism by SULT2B1‐KO. Tumour cells can exploit lipid metabolism to profit from the metastatic cascade, encompassing the genesis of metastasis‐initiating cells up to the eventual metastatic outgrowth. Research on SCD1 revolves around SCD1 and its association with the metastatic process through modifications in crucial lipid metabolism pathways. SCD1 inhibition alone or combined with inducing lipogenesis, emerges as a potential therapeutic strategy to boost lipotoxicity, leading to the demise of tumour cells.^50^, ^51^ Our research discovered that SULT2B1‐KO combined with CAY (SCD1 inhibitor) could maximise the inhibition of distant metastasis in vivo. As SULT2B1 could induce TG and TC accumulation in CC cells, we further speculated that blocking SULT2B1‐increased lipid metabolism may reduce distant metastasis. Statins are common lipid‐lowering drugs in clinical practice, such as lovastatin, pivastatin, simvastatin, pravastatin, fluvastatin, atorvastatin and rosuvastatin. Fluvastatin is less commonly used in clinical practice. And lovastatin, pivastatin, simvastatin and atorvastatin are metabolised through liver enzymes. Therefore, we selected the first generation of lovastatin, rosuvastatin and rosuvastatin to their role in CC metastasis. Through Transwell assays of CC cells, it was found that lovastatin and pravastatin can significantly suppress SULT2B1‐induced metastatic activity, and the effect of lovastatin is more obvious. Lovastatin has been reported to reduce serum triglyceride (TG) levels,^52^ inhibit cell proliferation,^53^, ^54^ reduce tumour metastasis,^55^, ^56^ as well as enhance anti‐tumour effects.^57^ Therefore, we further validated the effects of lovastatin on lipid metabolism and metastasis in vivo. In our study, it was demonstrated that lovastatin can block SULT2B1‐induced promotion effect of lipid metabolism and distant metastasis, indicating that lovastatin can be a potential target to reduce distant metastasis in CC patients with high expression of SULT2B1.

However, limitations of this research should be recognised. Although SULT2B1 showed significant facilitation of the metastasis of CC cells, the precise mechanisms through which SMC1A regulates SULT2B1 expression remain to be clarified in future studies. Moreover, prospective studies involving larger patient cohorts and extended follow‐up periods are encouraged to determine the prognostic value of SULT2B1 in CC.

In conclusion, our study demonstrated that SMC1A upregulated SULT2B1 expression, which interacted with SCD1 to modulate lipid metabolism, thereby increasing the metastatic capacity of CC (Figure 9). These findings offered novel insights into the mechanisms governing lipid metabolism regulation by SULT2B1 and its promotion of metastatic abilities in CC. Moreover, it provided a solid experimental basis for future clinical applications of targeted drugs that inhibited tumour cell lipid metabolism to suppress distant metastasis, as well as the potential use of lovastatin to reduce distant metastasis in CC patients with high expression of SULT2B1.

## 4 MATERIALS AND METHODS

### 4.1 Bioinformatics analysis

The scRNA‐seq data were obtained from GEO (Gene Expression Omnibus) database (normal: GSM4904237, GSM4904246, GSM4904240, GSM4994385; tumour: GSM4904234, GSM4904236, GSM4904239, GSM4904245, GSM4994386; liver metastasis: GSM6886540, GSM7058761, GSM7058760), which were analysed by Seurat (v3) R.^58^ Expression analysis, survival analysis and correlation analysis were performed via GEPIA^59^ and Assistant for Clinical Bioinformatics^35^ using The Cancer Genome Atlas (TCGA) data.^60^

### 4.2 CC specimens

From patients undergoing surgical procedures at the First Affiliated Hospital of Zhejiang University, we acquired 117 pairs of CC specimens alongside corresponding adjacent normal tissue samples. Table 1 lists the clinicopathological features of 117 patients. Ethics approval was provided by the Medical Ethics Committee of First Affiliated Hospital of Zhejiang University, following the ethical standards of Declaration of Helsinki (reference number: II20230370A).

**TABLE 1:** Baseline characteristics of patients with colon cancer.

	Count (*N* = 117)	
Characteristics	*N*	%
Sex		
Male	62	53
Female	55	47
Age (years)		
<60	70	59.8
≥60	47	40.2
TNM stage		
I	8	6.8
II	25	21.4
III	39	33.3
IV	45	38.5
SULT2B1 expression		
High	59	50.4
Low	58	49.6
Metastasis		
M0	77	65.8
M1	40	34.2

### 4.3 Proteomics analysis

Fresh postoperative specimens of patients who underwent surgery at the First Affiliated Hospital of Zhejiang University were collected for proteomics, including three primary tumour tissues (T1–T3) and adjacent tissues (N1–N3) from patients without distant metastasis, and three primary tumour tissues (LM1–LM3) and adjacent tissues (N4–N6) from patients with liver metastasis. The tissue samples underwent tandem mass tags (TMT) proteomic analysis (conducted by Luming Biotechnology). Subsequently, the tissues were extracted, digested and labelled using TMT, followed by preparation for detection through liquid chromatography‐mass spectrometry (LC‐MS). ProteomeDiscoverer (v.2.4) was used to search all of the raw data thoroughly against the Uniprot Homo sapiens database.

### 4.4 Cell culture and transfection

HEK‐293T, SW480 cells and HCT116 were procured from American Type Culture Collection. Cells were cultured and sustained in DMEM or McCoy's 5A culture medium with 10% FBS. siRNAs targeting SULT2B1, SMC1A and (SMC3) (Tsingke) were used for transient transfection. And plasmids containing full‐length cDNA of SULT2B1, SMC1A and SMC3 were used for transient overexpression transfection. CRISPR‐Cas9 editing system was employed to create SULT2B1‐KO cells in HCT116 cells based on the published protocol.^7^ Additionally, we generated the PCDH‐CMV‐MCS‐EF1‐GFP‐Puro vector (Tsingke) overexpressing SULT2B1. Overexpressing lentivirus particles were produced using HEK‐293 T cells for the stable transfection of HCT116 cells. Table 2 displays the siRNA and sgRNA sequences.

**TABLE 2:** Primer, siRNA and sgRNA sequences in the research.

Name	Sequence (5ʹ–3ʹ)
SULT2B1 forward	CGGGACGACGACATCTTTATC
SULT2B1 reverse	GCTGAAGGCACCCACAATG
GAPDH forward	GGAGCGAGATCCCTCCAAAAT
GAPDH reverse	GGCTGTTGTCATACTTCTCATGG
SCD1 forward	GCCCCTCTACTTGGAAGACGA
SCD1 reverse	AAGTGATCCCATACAGGGCTC
SULT2B1 CHIP Primer 1‐forward	AGATCAGCGCCATTTCCCAA
SULT2B1 CHIP Primer 1‐reverse	CTACCCACGCCTCCAACAG
SULT2B1 CHIP Primer 2‐forward	GTTGAGGATGTGGACTCGGT
SULT2B1 CHIP Primer 2‐reverse	TTGTTGCATAGGTGAGGGGG
si.SULT2B1‐1	CCAACACCATGTCCAACTA
si.SULT2B1‐2	GATCGAGATCATCTGCTTA
sg.RNA	GGGAGACCACAACGTCCCGGGGG

### 4.5 Transwell assay

For invasion assay, 4 × 10^4^ cells in FBS‐free culture medium were seeded into Transwell inserts precoated with 5% gelatin (50 μL/inserts; Sangon), and incubated for 24 h. The lower chambers were filled with complete culture medium containing 10% FBS.

For migration assay, the Transwell inserts were left uncoated. 2 × 10^4^ cells in FBS‐free culture medium were seeded into Transwell inserts for 24 h, and the lower chambers were supplemented with complete culture medium with 10% FBS.

### 4.6 RNA sequencing analysis

HCT116 cells were treated with si.NC, si.SULT2B1‐1 and si.SULT2B1‐2 for 48 h. After the treatments, total RNA was extracted from indicated HCT116 cells (si.NC, si.SULT2B1‐1, si.SULT2B1‐2) by TRIzol reagent (Invitrogen) following the manufacturer's protocol. The extracted RNA underwent assessment for purity, quantification, as well as integrity. Subsequently, the libraries were constructed employing VAHTS Universal V6 RNA‐seq Library Prep Kit, adhering to the manufacturer's guidelines. OE Biotech Co., Ltd. outsourced transcriptome sequencing and analysis. Clean reads were aligned to the human genome utilising HISAT. FPKM values for each gene were computed, and the read counts per gene were acquired via HTSeq count. Differential expression analysis was conducted employing DESeq, setting the threshold for DEGs at a *p*‐value less than 0.05 and a fold change greater than 1.5 or less than 0.5.

### 4.7 Oil Red O Staining kit

The cells were fixed and subsequently stained using an Oil Red O Staining kit following the guidelines provided by the manufacturer (B1094, Applygen Technologies Inc).

### 4.8 Triglyceride and cholesterol assay

Tissue and cell levels of triglycerides and total cholesterols (TC) were detected by TG (E1013, Applygen Technologies Inc) and TC testing kit (E1015, Applygen Technologies Inc) in accordance with the manufacturer's guidelines.

### 4.9 Lipidomic analysis

HCT116 cells were treated with si.NC, si.SULT2B1‐1 and si.SULT2B1‐2 for 48 h. Then, indicated HCT116 cells were collected for lipid extraction, which were then analysed by Thermo Scientific Dionex UltiMate 3000 HPLC system (Thermo Scientific) coupled with a Q Exactive hybrid quadrupole‐orbitrap mass spectrometer (Thermo Scientific). In the context of UHPLC–MS/MS analysis, lipid chromatographic separation was conducted employing the UHPLC‐Q Exactive HF‐X Vanquish Horizon system (Thermo Scientific) by Majorbio. Lipid species were identified and quantified using Lipid Search software.

### 4.10 Immunofluorescence

The comprehensive methodology for IF was extensively elucidated in prior study.^61^ Antibody used are displayed in Table 3. IF results were imaged using the confocal mode of IXplore SpinSR (100×/1.4 oil, Olympus Corporation). Data were processed using STEDYCON Gallery software.

**TABLE 3:** The primary antibody in this paper were as follows.

Name	Article numbers (brand)
SULT2B1	A7736 (ABCLONE)
SCD1	ab19862 (Abcam)
Phalloidin	PF00001 (proteintech)
GAPDH	60004‐1‐Ig (proteintech)
Ki67	27309‐1‐AP (proteintech)
SMC1A	21695‐1‐AP (proteintech)
SMC3	#5696S (CST)
secondary antibodies	#7074, #7076 (CST)
Fluorescent secondary antibody	SA00013‐4, SA00014‐10 (proteintech)

### 4.11 RNA extraction and RT‐qPCR analysis

The cell or tissue RNA was extracted utilising RNAiso Plus (TaKaRa), while the mRNA underwent reverse transcription employing the PrimeScript RT Reagent Kit with gDNA Eraser (TaKaRa) to generate first‐strand cDNA. Subsequent quantitative RT‐PCR analysis was conducted using the SYBR Premix Ex Taq (TaKaRa). Table 2 provides the primer sequences.

### 4.12 Western blotting

The cell or tissue extracts were obtained utilising RIPA lysis buffer (Beyotime) supplemented with a protease inhibitor, followed by Western blotting (WB) analysis. Details regarding the antibodies employed are enumerated in Table 3.

### 4.13 Immunohistochemistry

The detailed procedures of immunohistochemistry (IHC) were described previously.^62^ The results underwent semi‐quantitative analysis. Dye intensity determined the grading scale: absence of dyeing received a score of 0, faint yellow received 1, pale brown received 2 and brown received 3. Mean values across five visual fields (at ×400 magnification) were computed to ascertain the percentage of positive tumour cells. Scoring categorised the percentage of positive tumour cells as follows: 0 for visual fields with <1%, 1 for 1%–25%, 2 for 25%–75% and 3 for 75%–100%. The final score was derived by adding the dye intensity score to the positive cell score. Antibodies used here were listed in Table 3.

### 4.14 TUNEL assay

In situ cell death, paraffin‐embedded specimens were tested using in situ cell death detection kit (fluorescein method) (11684795910, Roche) following the guidelines of the manufacturer.

### 4.15 Immunoprecipitations (IP)

HCT116 cells whole‐cell protein was extracted by modified RIPA extraction buffer (Beyotime), which was then incubated at 4°C overnight with protein A/G‐agarose beads (Sigma Aldrich) and corresponding antibody, and the precipitates were washed five times with RIPA. Complexes were eluted by heating at 100°C for 10 min, then analysed using WB.

### 4.16 Immunoprecipitations mass spectrometry (IP‐MS)

Protein bands of immunoprecipitation were visualised with Coomassie Brilliant Blue stain (P0017, Beyotime). And in‐gel were digested for HPLC tandem mass spectrometry (LC/MS) by the First Affiliated Hospital of Zhejiang University. The resulting MS/MS raw data were processed using Proteome Discoverer software v2.5, developed by Thermo Scientific. Tandem mass spectra were searched against SwissProt Human database using the SEQUEST algorithm.

### 4.17 Pull‐down assay

For GST pull‐down assays, 2500 ng GST‐Overexpression plasmid of SULT2B1 and 10 μL lipo‐3000 (L3000015, Thermo Fisher Scientific) were added into a 10 cm dish of HCT116 cells. Following a 48‐h incubation period, cells were harvested, suspended in lysis buffer and subsequently sonicated. The resulting lysates underwent centrifugation, and the supernatants were applied to Glutathione Sepharose beads (NRPB56S, Nuptec) that had been equilibrated with lysis buffer at 4°C for 4 h. Subsequently, the beads were retrieved and subjected to three washes with lysis buffer. GST‐fusion proteins were then eluted from the beads using reduced glutathione. Following this elution, the adsorbed proteins were subjected to analysis via Western blot after a final washing step, and were visualised with Coomassie Brilliant Blue stain or Silver Staining (LC6070, Thermo Scientific).

### 4.18 Molecular docking analysis

3D structures of SULT2B1^27^ and SCD1^28^ were downloaded from the PDB protein database.^29^ And the structure of the SULT2B1‐SCD1 complex was modelled by HDOCK,^30^ which was then visualised via PYMOL software.^31^

### 4.19 In silico residue scanning

The SCD1 protein's affinity toward SULT2B underwent assessment through residue scanning within the MOE suite's protein design package.^63^ Employing unary quadratic optimisation (UQO) within the LowMode ensemble, the protein–protein complex underwent residue scanning. This technique utilised LowModeMD to explore the mutants' conformational space. The LowModeMD approach initiated mutant conformations via a brief, approximately 1 ps molecular dynamics (MD) simulation at a constant temperature, succeeded by all‐atom energy minimisation. Successful conformations meeting the required energetic and geometric criteria were stored in the output database. For accelerated simulation, atoms more than 5.0 Å distant from the ligand were designated as inert, with iterations capped at 50, and a limit of five conformations set for each mutated complex. Receptor SCD1 residues underwent mutation, and consequent changes in the binding affinity of the complex were recorded.

### 4.20 Double luciferase reporter gene experiment

1 × 10^5^ Cells were seeded per well in 12‐well plates. Co‐transfection involved 0.3 μg of SULT2B1‐promoter‐pGL3‐basic dual luciferase reporter plasmid (Tsingke) or vector‐pGL3‐basic control plasmid (Tsingke) along with 0.2 μg of pRL‐TK‐Renilla plasmid (Tsingke) for 2 days. Luciferase activity was measured using a Dual‐Luciferase Assay kit (Promega) and a full‐wavelength microplate reader (Varioskan Flash, Thermo Scientific) in accordance with the manufacturer's guidelines.

### 4.21 Chromatin immunoprecipitation assay (CHIP)

Detailed procedures of this assay were conducted followed the direction of a chip kit (P2080S, Beyotime). Briefly, 10 cm dish HCT116 cells were lysed, which were sonicated (10 s each time, three to four times in total, set to 30% of maximum power at 50 W). Then, the sheared chromatin was incubated at 4°C overnight with 50 μL Protein A/G Magnetic Beads/Salmon Sperm DNA and indicated 1 μg antibody. Bound DNA–protein complexes were eluted and digested with proteinase K, then the DNA was purified for PCR. And the products of PCR were then analysed by RT‐QPCR and DNA gel electrophoresis. Tables 2 and 3 listed the primer sequences and antibody.

### 4.22 Animal assays

The study involving animal experimentation obtained ethical approval (Reference Number: 2023−973) from the Committee of Animal Ethics at the First Affiliated Hospital of Zhejiang University. In an aseptic facility, female BLAB/c nude mice, aged 4–6 weeks, were bred. In our study, each experimental group comprised five mice that were randomly assigned, irrespective of the animal experiment group.

For lung metastases model, 1.0 × 10^6^ cells (HCT116 WT, HCT116 SULT2B1‐KO) were suspended in PBS in a volume of 150 μL, which were then injected through caudal vein to establish lung metastasis model.

In orthotopic model, 1.0 × 10^6^ cells (HCT116 WT [wild type], HCT116 SULT2B1‐KO [knock out]) and 5 × 10^5^ cells (HCT116 p.NC, HCT116 p.SULT2B1), suspended in a 1:1 mixture of PBS and Matrigel (356234, Corning) in a volume of 50 μL, were injected into the subserous layer in the mid‐region of the cecum in nude mice. SCD1 inhibitor‐CAY10566 (Sigma), which is abbreviated as CAY in this research, was used by oral administration every day (5 mg/kg) until death. And lovastatin (Sigma) was used by oral administration every day (20 mg/kg) until death. After 7 weeks, all mice were sacrificed, and lung and liver metastases were analysed.

### 4.23 Statistical analysis

The findings were displayed as the mean ± standard deviation (SD). Statistical comparisons were conducted utilising either the Student's *t*‐test or the nonparametric Mann–Whitney *U* test, denoting significance with a threshold of *p* < 0.05. SPSS Statistics software and GraphPad Prism 8 was used to carried out statistical analyses. Significance levels were symbolised as *p < 0.05, **p < 0.01 and ***p < 0.001.

## AUTHOR CONTRIBUTIONS

Gang Che, Wankun Wang and Jiawei Wang: writing—original draft, investigation, methodology, conceptualisation, formal analysis and data curation. Cheng He, Jie Yin and Zhendong Chen: investigation, visualisation and software. Chao He and Xujing Wang: tissue collection. Yan Yang and Jian Liu: funding acquisition, project administration, resources, supervision, writing—review, validation.

## CONFLICT OF INTEREST STATEMENT

The authors declare that they have no conflicts of interest.

## ETHICS STATEMENT

This study involving human tissue acquired ethics approval (Reference Number: II2030370A) from the Medical Ethics Committee of the First Affiliated Hospital of Zhejiang University. Additionally, all animal experiments received approval from the Committee of Animal Ethics of the same institution (Reference Number: 2023−973).

## Supporting information

Supporting Information

Supporting Information

Supporting Information

Supporting Information

Supporting Information

Supporting Information

Supporting Information

Supporting Information

## Data Availability

Sc‐RNA seq data of CC were downloaded from the GEO database (https://www.ncbi.nlm.nih.gov/gds/). CC cases were extracted from TCGA database (https://cancergenome.nih.gov/). Raw data of proteomics, RNA sequencing, non‐target lipidomics and IP‐MS analysis can be obtained from the corresponding author of article. Gene expression matrix of epithelial cells in scRNA‐seq data, as well as the outcomes from proteomics, including the corresponding DEGs or DEPs, were illustrated in Tables S1 S2 and S3, respectively.
