# Supplementary figures and images for: Sulfotransferase SULT2B1 facilitates colon cancer metastasis by promoting SCD1‐mediated lipid metabolism
Source: Clin Transl Med. 2024 Feb 19;14(2):e1587. doi: 10.1002/ctm2.1587 (PMC10875708; doi:10.1002/ctm2.1587)

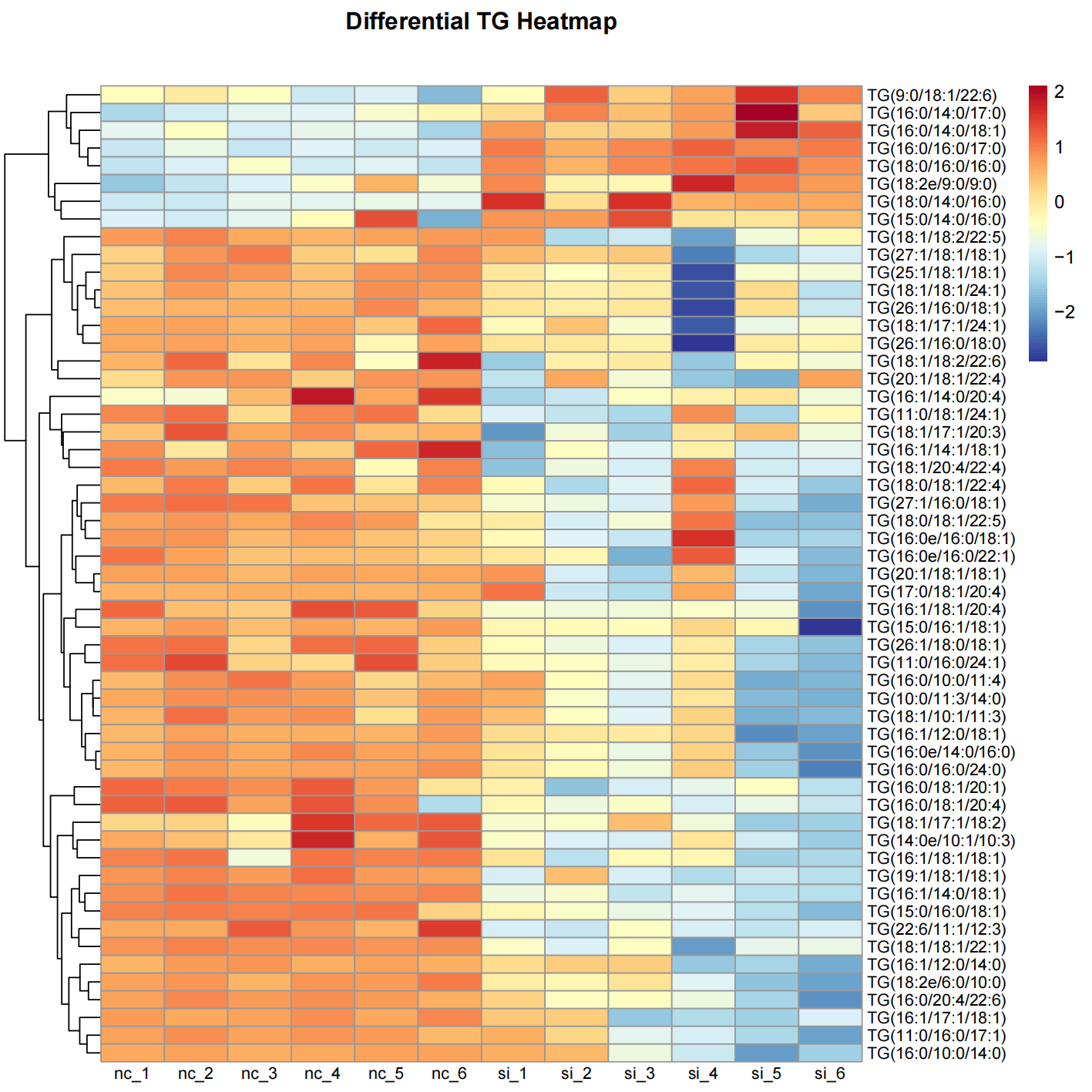

Supplement: Supplementary file 1 — Supporting Information [file CTM2-14-e1587-s001.tif]

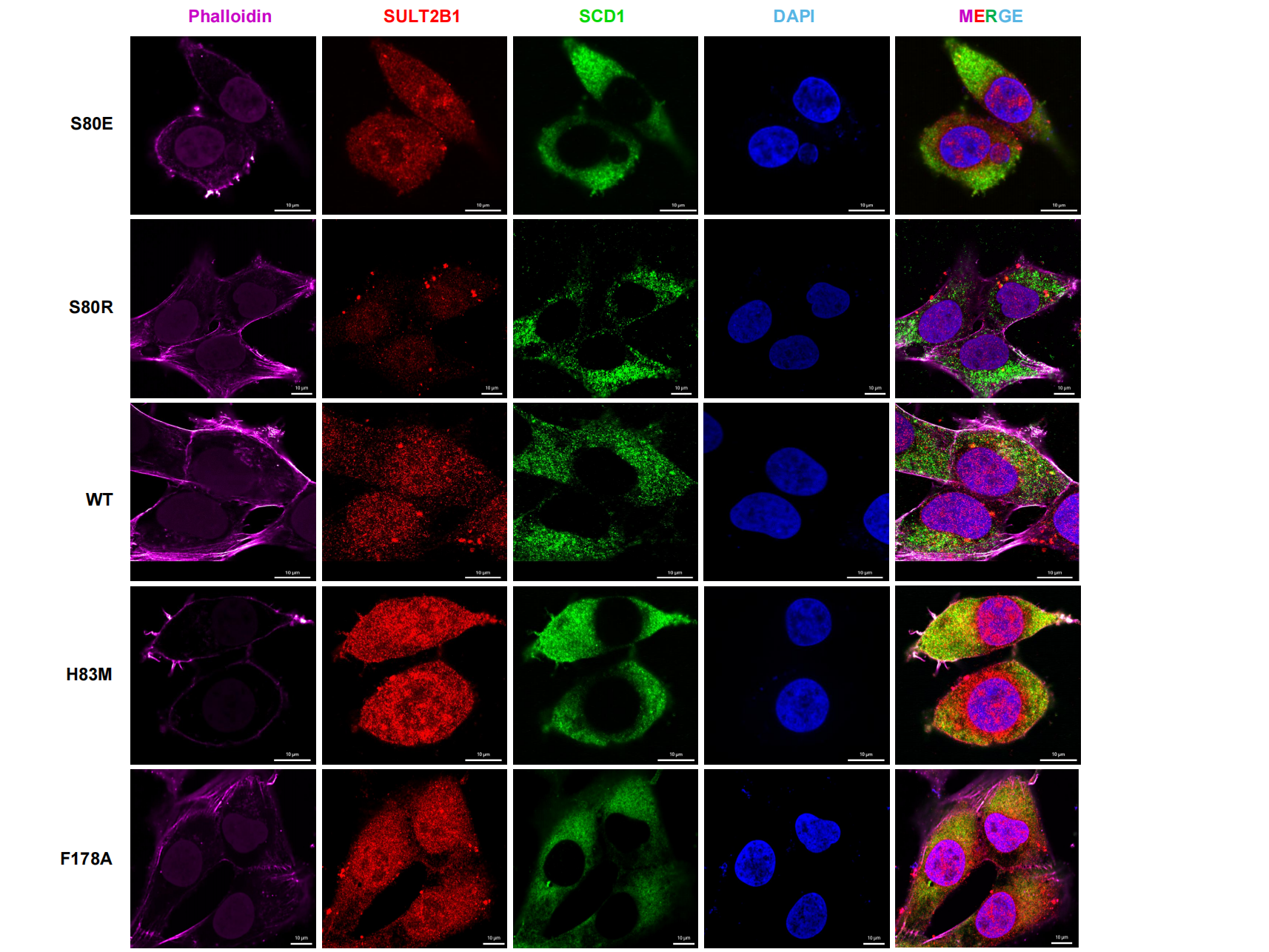

Supplement: Supplementary file 2 — Supporting Information [file CTM2-14-e1587-s008.tif]

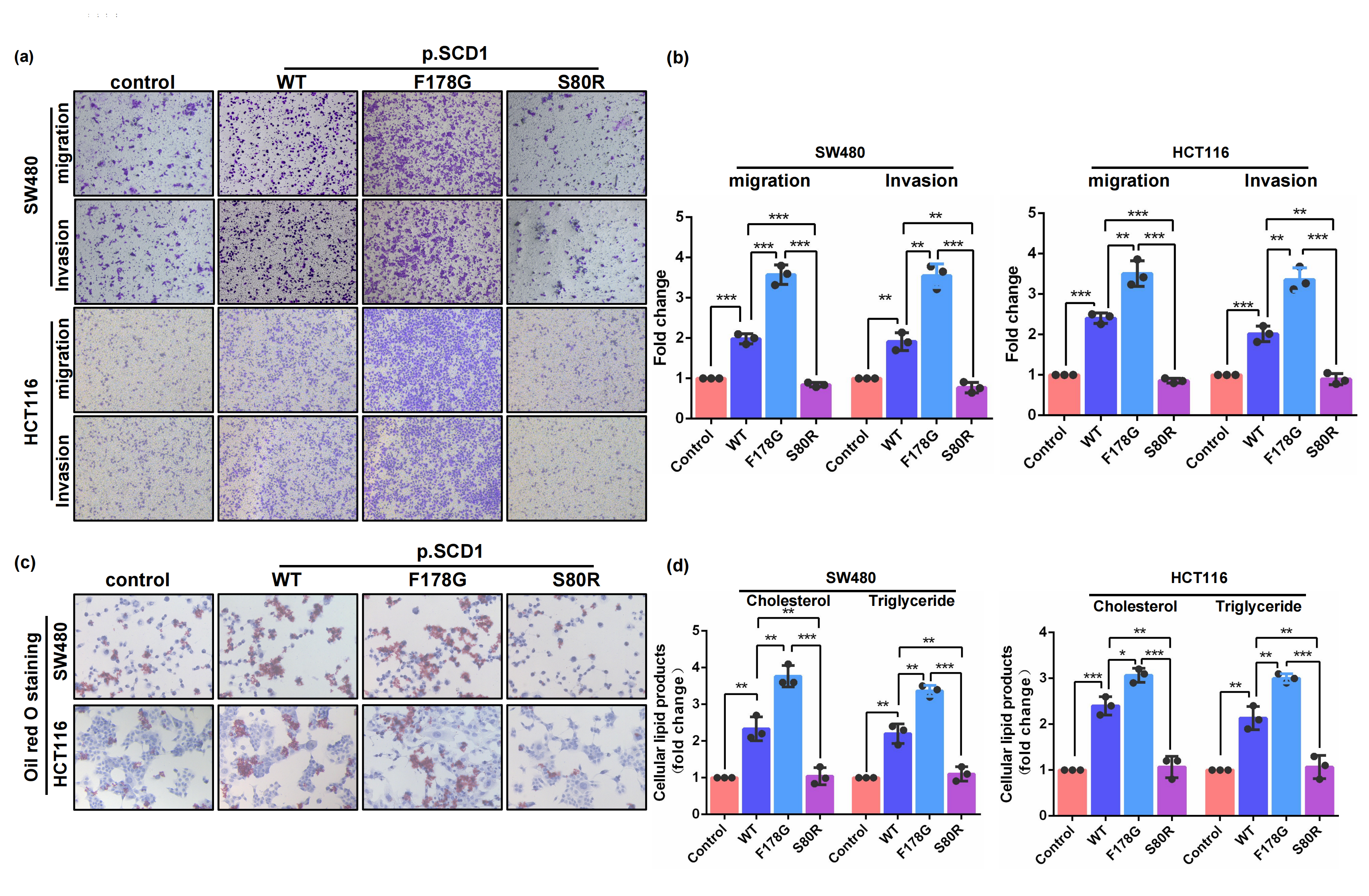

Supplement: Supplementary file 3 — Supporting Information [file CTM2-14-e1587-s002.tif]

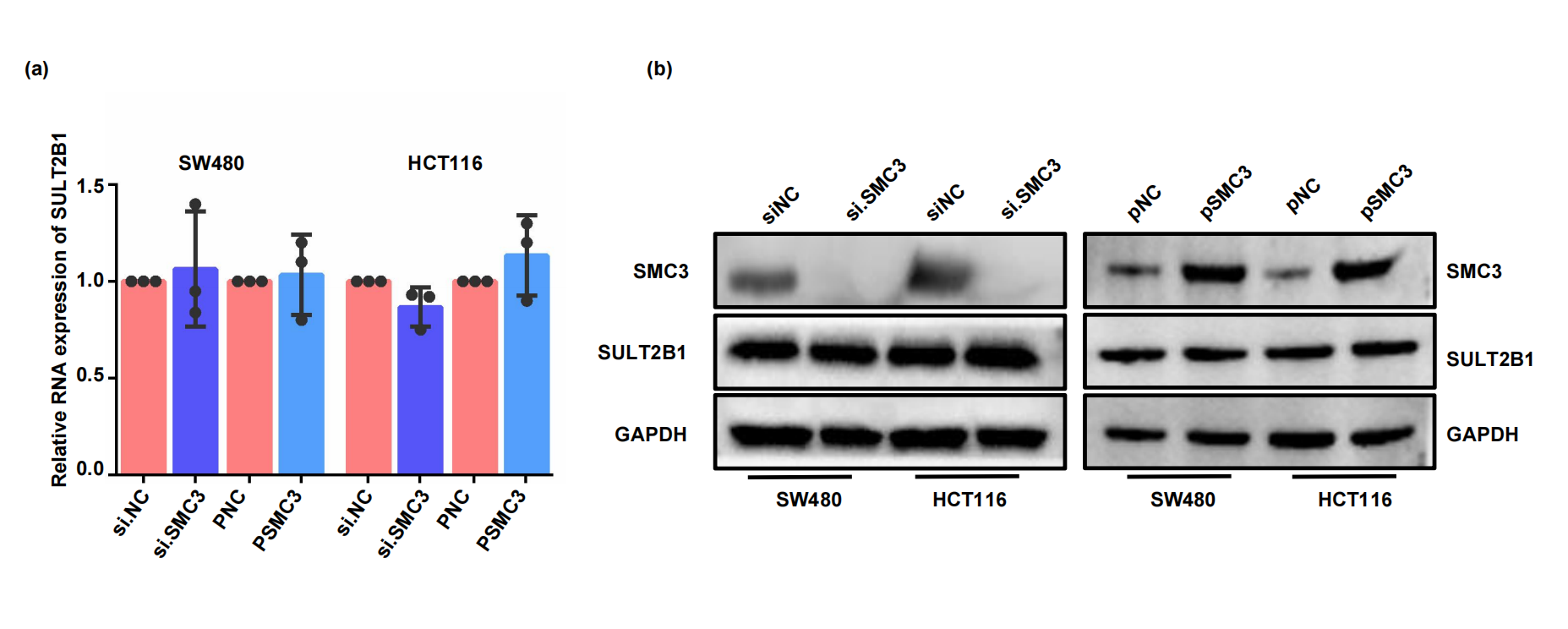

Supplement: Supplementary file 4 — Supporting Information [file CTM2-14-e1587-s003.tif]

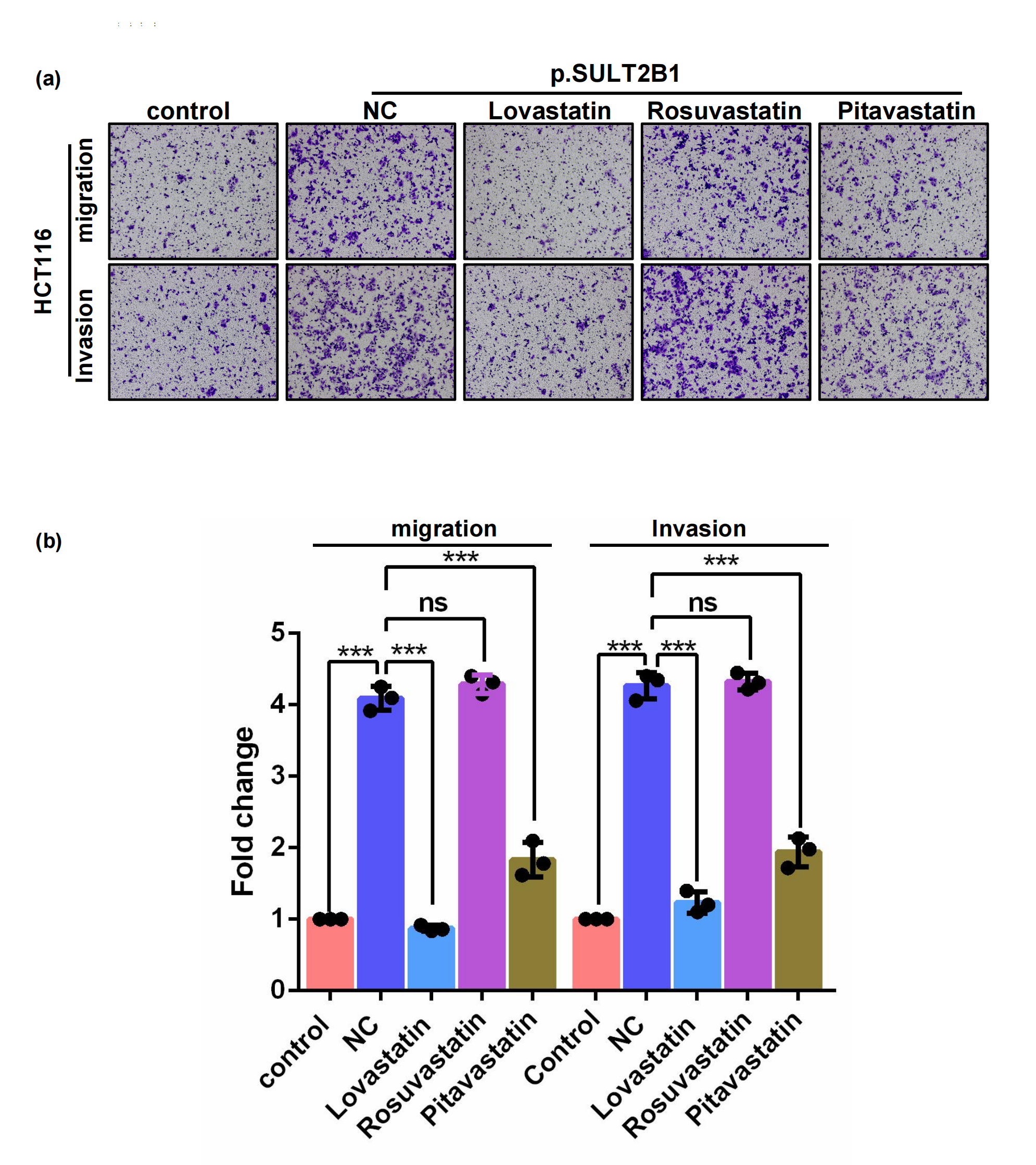

Supplement: Supplementary file 5 — Supporting Information [file CTM2-14-e1587-s005.tif]
